# Preoperative Platelet-Lymphocyte Ratio (PLR) as a prognostic inflammation biomarker in Asian HIV-infected patients with gastric cancer: a single-center study

**DOI:** 10.1186/s12876-023-02828-x

**Published:** 2023-05-26

**Authors:** Li Deng, Lei Li, Yiling Qiu, Ye Cao, Shixian Lian, Yanhui Si

**Affiliations:** 1 Department of General Surgery, Shanghai Public Health Clinical Center, Fudan University, Shanghai, China; 2 Department of Infectious Diseases, Children’s Hospital of Fudan University, Shanghai, China

**Keywords:** HIV, Gastric cancer, Systemic inflammation biomarker, Platelet to lymphocyte ratio, Prognosis

## Abstract

**Background:**

The serum systemic inflammation biomarkers have been established as predictors of prognosis in gastric cancer (GC) patients, but their prognostic value in human immunodeficiency virus (HIV)-infected patients with GC has not been well studied. This retrospective study aimed to evaluate the prognostic value of preoperative systemic inflammation biomarkers in Asian HIV-infected patients with GC.

**Methods:**

We retrospectively analyzed 41 HIV-infected GC patients who underwent surgery between January 2015 and December 2021 at the Shanghai Public Health Clinical Center. Preoperative systemic inflammation biomarkers were measured and patients were divided into two groups based on the optimal cut-off value. Overall survival (OS) and progression-free survival (PFS) were measured using the Kaplan–Meier method and the log-rank test. Multivariate analysis of variables was performed using the Cox proportional regression model. As a comparison, 127 GC patients without HIV infection were also recruited.

**Results:**

The median age of the 41 patients included in the study was 59 years, with 39 males and two females. The follow-up period for OS and PFS ranged from 3 to 94 months. The cumulative three-year OS rate was 46.0%, and the cumulative three-year PFS rate was 44%. HIV-infected GC patients had worse clinical outcomes compared to the normal GC population. The optimal cut-off value for preoperative platelet to lymphocyte ratio (PLR) was 199 in HIV-infected GC patients. Multivariate Cox regression analysis revealed that a low PLR was an independent predictor of better OS and PFS (OS: HR = 0.038, 95% CI: 0.006–0.258, *P* < 0.001; PFS: HR = 0.027, 95% CI: 0.004–0.201, *P* < 0.001). Furthermore, higher preoperative PLR in HIV-infected GC was significantly associated with lower BMI, hemoglobin, albumin, CD4 + T, CD8 + T, and CD3 + T cell counts.

**Conclusion:**

The preoperative PLR is an easily measurable immune biomarker that may provide useful prognostic information in HIV-infected GC patients. Our findings suggest that PLR could be a valuable clinical tool for guiding treatment decisions in this population.

**Supplementary Information:**

The online version contains supplementary material available at 10.1186/s12876-023-02828-x.

## Introduction

Gastric cancer (GC) remains a major cause of cancer-related mortality globally [1]. While D2 gastrectomy and chemotherapy are the standard treatment, the prognosis for advanced stages is still unsatisfactory [2, 3]. In recent years, there has been an increase in GC cases among patients infected with the human immunodeficiency virus (HIV) [4, 5]. *H.pylori* infection is a significant risk factor for GC, particularly in East-Asia, and HIV-induced immunodeficiency is thought to exacerbate this risk [4, 5]. Furthermore, HIV infection impairs the HIV-specific CD4 + T and CD8 + T lymphocytes, resulting in the inability of the host immune system to control HIV, which may lead to poorer outcomes of HIV-related cancers [6]. Early identification of the worse prognostic subgroup of HIV-infected GC is crucial for patients' survival [7].

Although molecular subtypes had the potential to identify GC patients with a worse prognosis, the results based on genomic data made them difficult to use in clinical practice. In addition to cancer-associated systemic inflammation, which is an important predictor of prognosis in GC patients, systemic inflammation biomarkers such as the neutrophil to lymphocyte ratio (NLR), lymphocyte to monocyte ratio (LMR), platelet to lymphocyte ratio (PLR), prognostic nutritional index (PNI), systemic immune-inflammation index (SII), and systemic inflammation score (SIS) have been shown to be useful prognostic factors in numerous studies [8–11]. Building on previous research that suggests combination antiretroviral therapy (cART) can restore immune function in HIV-infected individuals [12] and that there may be no difference in tumor-infiltrating lymphocytes between HIV-positive and HIV-negative cancer [13], it may be feasible to evaluate systemic inflammatory biomarkers in HIV-infected GC patients. However, to our knowledge, no research has been conducted on the relationship between preoperative systemic inflammation biomarkers and HIV-infected GC. Early identification of these clinically feasible predictors could improve patients’ chances of survival. Therefore, this study aims to evaluate the prognostic value of systemic inflammation biomarkers in HIV-related GC patients.

## Methods

### Patients

We conducted a retrospective study of 41 HIV-infected patients with preoperative pathologically diagnosed gastric adenocarcinoma who underwent gastrectomy (with or without standard lymphadenectomy) between January 2015 and December 2021 at Shanghai Public Health Clinical Center (SHPHC) in China. The study included characteristics such as gender, age, body mass index (BMI), disease history (hypertension, diabetes, tuberculosis infection, smoking, and alcohol abuse), duration of HIV infection and treatment (cART), preoperative tests (routine laboratory tests, tumor markers, and plasma lipids), tumor location, pathological stage (using the AJCC 8th edition), human epidermal growth factor receptor 2 (Her2) status (using immunohistochemical results of Her2 proteins), and surgical resection with or without tumor residuals. For comparison, we also retrospectively recruited 127 non-HIV infected patients diagnosed with gastric adenocarcinoma who underwent surgery treatment in our hospital during the same period. This study was conducted in accordance with the Declaration of Helsinki and approved by the Shanghai Public Health Clinical Center.

### Follow-up

Regular follow-ups were conducted every three months during the first year after surgery and every six months for the following two years. Patients were admitted to the hospital for routine blood examination, enhanced chest and abdominal CT scans, and other necessary tests. After three years, the follow-up visits were scheduled annually. Progression was defined as the first detection of local recurrence, enlargement of unresected tumor lesions, and distant organ metastases. Death was confirmed through relevant information provided by the hospital or by notification from the patient's family during a telephone follow-up. The last censoring date for evaluating survival time was March 2022. Overall survival (OS) was defined as the time interval from surgery to death from any cause or the last censoring date. Progression-free survival (PFS) was defined as the time interval from surgery to progression or the last censoring date. Two of the 41 patients were lost to follow-up during the study period.

### Definition of inflammation-based biomakers

The laboratory tests from each patient were obtained within 1-week before surgical resection of the primary tumour. In recent reports, neutrophil (N; × 10^9/L) to lymphocyte (L; × 10^9/L) ratio (NLR), lymphocyte (L; × 10^9/L) to monocyte (M; × 10^9/L) ratio (LMR) and platelet (P; × 10^9/L) to lymphocyte (L; × 10^9/L) ratio (PLR) had been evaluated as useful prognostic biomarkers of GC patients [14, 15]. The prognostic nutritional index (PNI) was calculated as serum albumin value (g/L) + 5 × lymphocyte (L; × 10^9/L) [16]. The systemic immune-inflammation index (SII) was calculated based on platelet (P; × 10^9/L), neutrophil (N; × 10^9/L), and lymphocyte (L; × 10^9/L) blood counts using the following formula: SII = P × N/L [17]. The systemic inflammation score (SIS) definition was based on the combination of the preoperative albumin level and LMR [9]. The SIS was defined as follows: patients with albumin level < 40 g/L and LMR < 4.44 were assigned a score of 2; patients with either albumin level ≥ 40 g/L or LMR ≥ 4.44 were assigned a score of 1; and patients with both albumin level ≥ 40 g/L and LMR ≥ 4.44 were assigned a score of 0.

### Statistics

Categorical variables were reported as integers and proportions, and continuous variables were reported as medians, means (standard deviations), and maximum ranges. We used Wilcoxon rank-sum test for continuous variables and chi-square for classified variables. To analyze the survival differences, patients were categorized into two group based on the continuous variables best-cutoff using the “MaxStat” R package (maximally selected log-rank statistics) [18]. The Kaplan–Meier survival curves were constructed according to differences in group, which were analyzed using the log-rank test. Variables that significantly affected survival were estimated by univariate and multivariate analysis according to the Cox regression model. Stratifified survival analysis results were discussed based on clinicopathological features. A two-tailed *P* < 0.05 was considered statistically significant. All statistical analyses were determined using R software (version 3.6.3, http://www.r-project.org).

## Results

### Patient characteristics

The clinicopathological characteristics of 41 HIV-infected patients with gastric cancer (GC) who underwent gastrectomy are summarized in Table S1. Of the 41 patients, 39 were male and two were female, with a median age of 59 years (range: 44–76 years) at the time of surgery. The median body mass index (BMI) was 21.1 (range: 15.1–26.6), and 22.0% of patients had a history of hypertension, 17.1% had diabetes, 4.9% had tuberculosis infection, and 17.1% had hyperlipidemia. Eight patients (19.5%) had a history of smoking, and five (12.2%) had a history of alcohol abuse. The median duration of HIV infection was one month, and most patients had not received anti-HIV treatment. The preoperative test showed a mean CD4 + T lymphocyte count of 323.4 cell/µL and a CD8 + T lymphocyte count of 663.3 cell/µL. Based on postoperative pathology, 7 patients (17.1%) had an upper gastric tumor, 31 (75.6%) had a middle gastric tumor, and 3 (7.3%) had a tumor in the lower part of the stomach. According to the eighth edition of the AJCC TNM staging system, 9 (22.0%), 8 (19.5%), 19 (46.3%), and 5 (12.2%) of patients had stages I, II, III, and IV, respectively.

### Survival outcomes

**Fig. 1:**
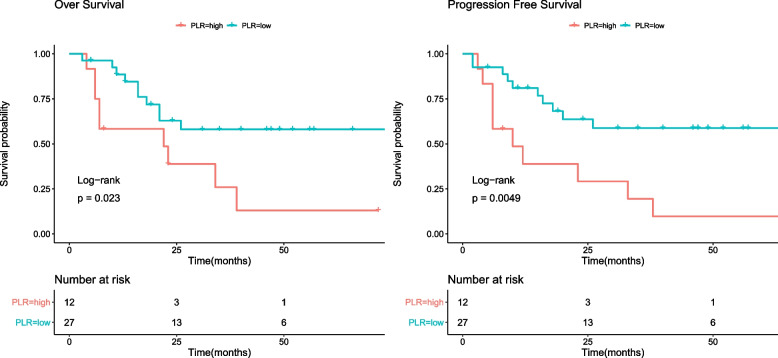
The Kaplan–Meier survival analysis of overall survival and progression free survival according to PLR in HIV-related GC

**Fig. 2:**
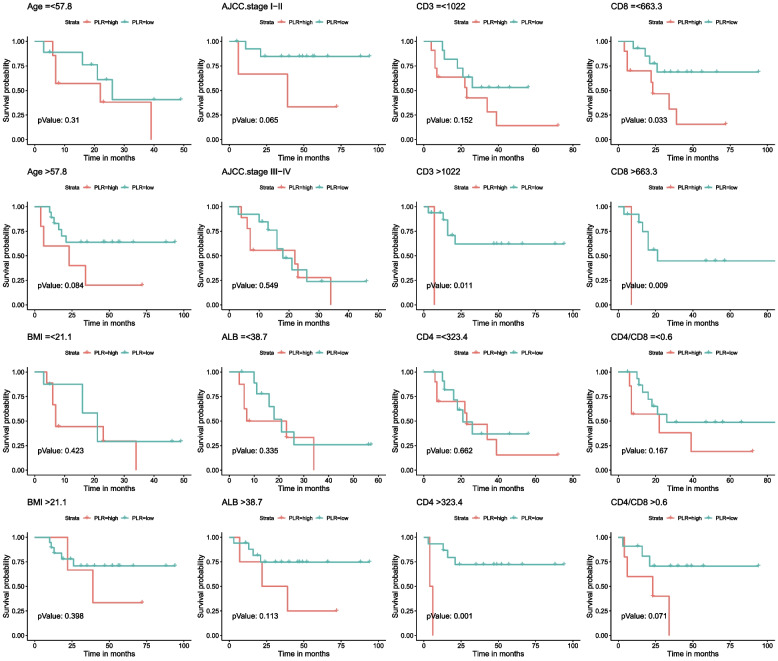
Subgroup analysis of OS based on age, BMI, AJCC stage, albumin level, CD3 cell counts, CD4 cell counts, CD8 cell counts and CD4/CD8 ratio

**Fig. 3:**
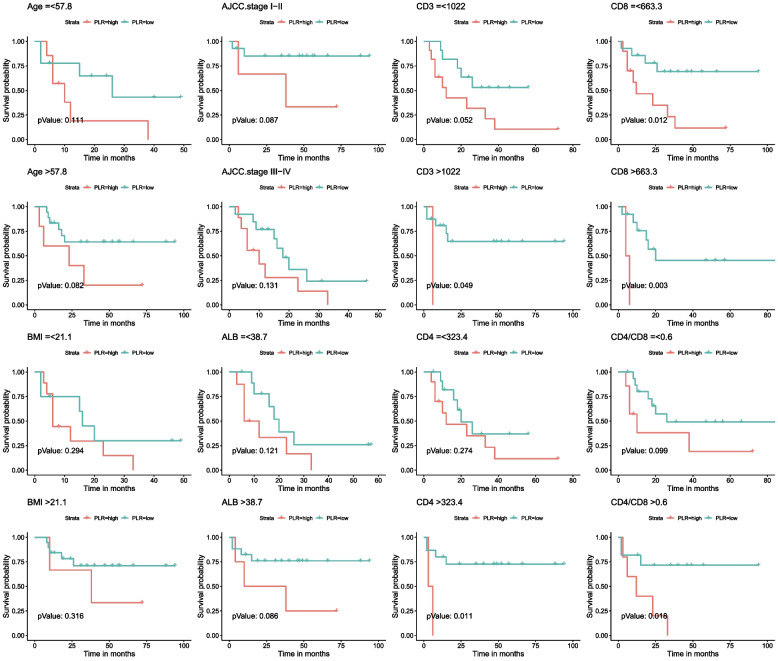
Subgroup analysis of PFS based on age, BMI, AJCC stage, albumin level, CD3 cell counts, CD4 cell counts, CD8 cell counts and CD4/CD8 ratio

The follow-up time for overall survival (OS) and progression-free survival (PFS) ranged from 3 to 94 months. The cumulative three-year OS rate was 46.0%, and the cumulative three-year PFS rate was 44%. HIV-infected GC patients had significantly worse OS and PFS outcomes compared to non-HIV infected GC patients (Figure S1). Univariate analysis identified several inflammatory indicators associated with OS, including PLR, PNI, and Alb level (*P* < 0.05, Table 1). Variables such as BMI, history of signet ring cell, R0 resection, and TNM stage also significantly affected OS (*P* < 0.05, Table 1). In multivariate analyses, PLR ≤ 199.0 (HR: 0.038, 95% CI: 0.006–0.258), histology of signet ring cell (HR: 41.178, 95% CI: 6.287–269.722), and no R0 resection (HR: 277.461, 95% CI: 10.475–7349.318) were associated with OS (both *P* < 0.05, Table 1). Additionally, the prognostic effect of NLR, PLR, PNI, SII, BMI, histology of signet ring cell, R0 resection, TNM stage, and Alb level was significantly related to PFS in univariate analysis (Table 2). In multivariate analysis of PFS, PLR ≤ 199.0 (HR: 0.027, 95% CI: 0.004–0.201), histology of signet ring cell (HR: 42.638, 95% CI: 6.245–291.092), and no R0 resection (HR: 136.565, 95% CI: 8.048–2317.405) were independently associated with PFS outcomes (both *P* < 0.05, Table 2). In non-HIV infected GC patients, inflammatory indicators such as PLR were not associated with OS (Table S2) or PFS (Table S3) in univariate analysis. The Kaplan–Meier survival analysis and log-rank tests were conducted to investigate the association between PLR groups and postoperative survival time. Our results showed that PLR (> 199) was significantly associated with decreased OS and PFS (Fig. 1). We also evaluated the OS and PFS subgroups of HIV-infected GC patients. The Kaplan–Meier curves revealed that CD3 + count > 1022 cells/µL, both CD8 + groups, and CD4 + count > 323.4 cells/µL in the high PLR group (> 199) were closely associated with poor OS (Fig. 2). Additionally, CD3 + count > 998.8 cells/µL, both CD8 + groups, CD4 + count > 291.3 cells/µL, and CD4/CD8 > 0.6 in the high PLR group (> 199) were also associated with poor PFS rate (Fig. 3).

**Table 1:** Univariate and multivariate analysis of clinicopathologic variables in relation to OS in patients living with HIV for GC

Clinicopathological features	Univariate analysis		Multivariate analysis	
	HR (95% CI)	*P* value	HR (95% CI)	*P* value
**Gender**				
Female	Reference			
Male	0(0-Inf)	0.998		
**Age**	0.973(0.915–1.033)	0.368		
**BMI**	0.763(0.634–0.918)	0.004	0.799(0.59–1.082)	0.147
**Hypertension**				
Yes	0.408(0.094–1.771)	0.231		
**DM**				
Yes	1.497(0.496–4.52)	0.474		
**TB**				
Yes	0.962(0.127–7.268)	0.97		
**Smoking**				
Yes	0.195(0.026–1.461)	0.112		
**Alcohol abuse**				
Yes	0.44(0.059–3.31)	0.425		
**Hyperlipidemia**				
Yes	0.763(0.222–2.622)	0.668		
**Duration of HIV infection**	1.001(0.988–1.014)	0.877		
**Duration of HIV treatment**	1.001(0.988–1.014)	0.882		
**Tumor Location**				
Upper	Reference			
Middle	0.892(0.256–3.111)	0.858		
Lower	1.609(0.267–9.696)	0.604		
**Histology**				
Signet ring cell	5.227(2.013–13.573)	< 0.001	41.178(6.287–269.722)	< 0.001
**R0 rescetion**				
No	5.204(1.678–16.141)	0.004	277.461(10.475–7349.318)	< 0.001
**Her2 status**				
0	Reference			
1 +	0.728(0.273–1.941)	0.525		
2 +	0.256(0.033–1.977)	0.192		
**AJCC stage**				
I-II	Reference		Reference	
III-IV	6.199(1.922–19.996)	0.002	0.314(0.034–2.867)	0.305
**ALB (g/L)**				
> 38.1	Reference		Reference	
≤ 38.1	4.192(1.599–10.991)	0.004	10.715(0.632–181.541)	0.1
**CD8 count (per ul)**				
> 661	Reference			
≤ 661	0.603(0.24–1.514)	0.281		
**CD4 count (per ul)**				
> 348	Reference			
≤ 348	2.646(0.943–7.425)	0.065		
**CD4/CD8**				
> 0.71	Reference			
≤ 0.71	2.062(0.738–5.762)	0.167		
**NLR**				
> 3.9	Reference			
≤ 3.9	0.416(0.157–1.102)	0.078		
**LMR**				
> 6.5	Reference			
≤ 6.5	3.325(0.442–24.99)	0.243		
**PLR**				
> 199.0	Reference		Reference	
≤ 199.0	0.365(0.147–0.903)	0.029	0.038(0.006–0.258)	< 0.001
**PNI**				
> 43.4	Reference		Reference	
≤ 43.4	3.05(1.216–7.653)	0.017	0.261(0.016–4.356)	0.349
**SII**				
> 935	Reference			
≤ 935	0.356(0.126–1.006)	0.051		
**SIS**				
0	Reference			
1	0.704(0.198–2.502)	0.587		
2	1.854(0.566–6.074)	0.308		

*DM* diabetes mellitus, *TB* tuberculosis infection status, *Her2 status* immunohistochemical staining of Her-2, *ALB* serum albumin, *NLR* neutrophil–lymphocyte ratio, *LMR* lymphocyte-monocyte ratio, *PLR* platelet-lymphocyte ratio, *PNI* prognostic nutritional index, *SII* systemic immune-inflammation index, *SIS* systemic inflammation score

**Table 2:** Univariate and multivariate analysis of clinicopathologic variables in relation to PFS in patients living with HIV for GC

Clinicopathological features	Univariate analysis		Multivariate analysis	
	HR (95% CI)	*P* value	HR (95% CI)	*P* value
**Gender**				
Female	Reference			
Male	0(0-Inf)	0.998		
**Age**	0.961(0.904–1.02)	0.192		
**BMI**	0.763(0.641–0.909)	0.002	0.884(0.684–1.142)	0.346
**Hypertension**				
Yes	0.363(0.084–1.572)	0.176		
**DM**				
Yes	1.282(0.428–3.839)	0.657		
**TB**				
Yes	0.824(0.109–6.21)	0.851		
**Smoking**				
Yes	0.177(0.024–1.325)	0.092		
**Alcohol abuse**				
Yes	0.399(0.053–2.991)	0.371		
**Hyperlipidemia**				
Yes	0.805(0.235–2.753)	0.729		
**Duration of HIV infection**	1.003(0.992–1.015)	0.586		
**Duration of HIV treatment**	1.003(0.991–1.016)	0.58		
**Tumor Location**				
Upper	Reference			
Middle	0.635(0.208–1.935)	0.424		
Lower	1.14(0.208–6.247)	0.88		
**Histology**				
Signet ring cell	3.939(1.578–9.836)	0.003	42.638(6.245–291.092)	< 0.001
**R0 rescetion**				
No	4.245(1.382–13.038)	0.012	136.565(8.048–2317.405)	< 0.001
**Her2 status**				
0	Reference			
1 +	0.806(0.302–2.149)	0.667		
2 +	0.563(0.126–2.521)	0.453		
**AJCC stage**				
I-II	Reference		Reference	
III-IV	6.297(1.96–20.228)	0.002	0.766(0.135–4.341)	0.764
**ALB (g/L)**				
> 38.1	Reference		Reference	
≤ 38.1	4.041(1.553–10.515)	0.004	3.73(0.309–45.103)	0.301
**CD8 count (per ul)**				
> 661	Reference			
≤ 661	0.741(0.302–1.822)	0.514		
**CD4 count (per ul)**				
> 348	Reference			
≤ 348	2.667(0.957–7.436)	0.061		
**CD4/CD8**				
> 0.71	Reference			
≤ 0.71	1.52(0.581–3.978)	0.394		
**NLR**				
> 3.9	Reference		Reference	
≤ 3.9	0.346(0.137–0.873)	0.025	0.109(0.008–1.56)	0.103
**LMR**				
> 6.5	Reference			
≤ 6.5	3.538(0.472–26.497)	0.219		
**PLR**				
> 199.0	Reference		Reference	
≤ 199.0	0.298(0.123–0.723)	0.007	0.027(0.004–0.201)	< 0.001
**PNI**				
> 43.4	Reference		Reference	
≤ 43.4	3.129(1.265–7.737)	0.014	0.756(0.057–10.062)	0.832
**SII**				
> 935	Reference		Reference	
≤ 935	0.275(0.104–0.729)	0.009	14.322(0.783–261.811)	0.073
**SIS**				
0	Reference			
1	0.555(0.156–1.978)	0.364		
2	1.667(0.515–5.393)	0.393		

*DM* diabetes mellitus, *TB* tuberculosis infection status, *Her2 status* immunohistochemical staining of Her-2, *ALB* serum albumin, *NLR* neutrophil–lymphocyte ratio, *LMR* lymphocyte-monocyte ratio, *PLR* platelet-lymphocyte ratio, *PNI* prognostic nutritional index, *SII* systemic immune-inflammation index, *SIS* systemic inflammation score

### The relationship between PLR and clinical characteristics

The relationship between clinicopathological variables and PLR was summarized in Table 3. Higher PLR values were associated with lower BMI (19.7 vs 21.7, *P* = 0.005), a longer duration of HIV treatment (32.2 months vs 20.9 months, *P* = 0.113), and a higher tendency for advanced stages (III-IV: 76.9% vs 50.0%, *P* = 0.173). The comparison of PLR with preoperative laboratory tests was shown in Table 4. In addition to platelet and lymphocyte counts, mean values of hemoglobin (126.9 g/L vs 104.2 g/L, *P* = 0.008), albumin (39.7 g/L vs 36.5 g/L, *P* = 0.041), CD3 + cells count (1196.1 per uL vs 646.9 per uL), CD4 + cells count (373 per uL vs 216.5 per uL), and CD8 + cells counts (784.2 per uL vs 402.8 per uL) were significantly higher in the low PLR group than in the high PLR group. However, neutrophil counts were significantly higher in the high PLR group (4.7 × 10^3/uL) than in the low PLR group (3 × 10^3/uL).

**Table 3:** Relationship between PLR and clinicopathological features in GC patients living with HIV

	low PLR group (*n* = 28)	high PLR group (*n* = 13)	*P*value
**Gender**			1
Male	26 (92.9%)	13 (100.0%)	
Female	2 (7.1%)	0 (0.0%)	
**Age**			0.408
Mean (SD)	58.2 (7)	56.8 (9.8)	
Median [MIN, MAX]	60 [44,70]	56 [44,76]	
**BMI**			0.005
Mean (SD)	21.7 (2.7)	19.7 (1.4)	
Median [MIN, MAX]	21.1 [15.1,26.6]	20.2 [17.3,21.7]	
**Hypertension**			0.692
No	21 (75.0%)	11 (84.6%)	
Yes	7 (25.0%)	2 (15.4%)	
**DM**			0.399
No	22 (78.6%)	12 (92.3%)	
Yes	6 (21.4%)	1 (7.7%)	
**TB**			1
No	26 (92.9%)	13 (100.0%)	
Yes	2 (7.1%)	0 (0.0%)	
**Smoking**			0.398
No	21 (75.0%)	12 (92.3%)	
Yes	7 (25.0%)	1 (7.7%)	
**Alcohol abuse**			1
No	24 (85.7%)	12 (92.3%)	
Yes	4 (14.3%)	1 (7.7%)	
**Hyperlipidemia**			1
No	23 (82.1%)	11 (84.6%)	
Yes	5 (17.9%)	2 (15.4%)	
**Duration of HIV infection**			0.377
Mean (SD)	22.7 (40.8)	32.5 (37.6)	
Median [MIN, MAX]	1 [0,132]	24 [0,108]	
**Duration of HIV treatment**			0.113
Mean (SD)	20.9 (37.2)	32.2 (37.2)	
Median [MIN, MAX]	0 [0,110]	24 [0,108]	
**Tumor Location**			1
Upper	5 (17.9%)	2 (15.4%)	
Middle	21 (75.0%)	10 (76.9%)	
Lower	2 (7.1%)	1 (7.7%)	
**Histology**			0.719
Signet ring cell	9 (32.1%)	3 (23.1%)	
**Her2 status**			0.66
0	13 (46.4%)	8 (61.5%)	
1 +	11 (39.3%)	3 (23.1%)	
2 +	4 (14.3%)	2 (15.4%)	
**SBR**			0.694
1	3 (10.7%)	2 (15.4%)	
2	8 (28.6%)	2 (15.4%)	
3	17 (60.7%)	9 (69.2%)	
**R0**			1
Yes	25 (89.3%)	12 (92.3%)	
No	3 (10.7%)	1 (7.7%)	
**T stage**			0.657
I	7 (25.0%)	2 (15.4%)	
II	1 (3.6%)	1 (7.7%)	
III	11 (39.3%)	4 (30.8%)	
IV	9 (32.1%)	6 (46.2%)	
**N stage**			0.719
Non-metastasis	9(32.1%)	3(23.1%)	
Metastasis	19(67.9%)	10(76.9%)	
**M stage**			0.645
Non-metastasis	25 (89.3%)	11 (84.6%)	
Metastasis	3 (10.7%)	2 (15.4%)	
**AJCC stage**			0.173
I-II	14(50.0%)	3(23.1%)	
III-IV	14(50.0%)	10(76.9%)	

*DM* diabetes mellitus, *TB* tuberculosis infection status, *Her2 status* immunohistochemical staining of Her-2, *R0* radical resection, *SBR* Scarff-Bloom-Richardson score, *PLR* platelet-lymphocyte ratio

**Table 4:** Relationship between PLR and preoperative laboratory tests in GC patients living with HIV

	Low PLR group (*n* = 28)	High PLR group (*n* = 13)	*P*value
**CA125 (0-35U/ml)**			0.232
Normal	27 (96.4%)	11 (84.6%)	
Increased	1 (3.6%)	2 (15.4%)	
**CA153 (0–32.3U/ml)**			0.317
Normal	28 (100.0%)	12 (92.3%)	
Increased	0 (0.0%)	1 (7.7%)	
**CA199 (0-37U/ml)**			0.228
Normal	20 (71.4%)	12 (92.3%)	
Increased	8 (28.6%)	1 (7.7%)	
**AFP (0.89–8.78 ng/ml)**			0.539
Normal	25 (89.3%)	13 (100.0%)	
Increased	3 (10.7%)	0 (0.0%)	
**CEA (0-5 ng/ml)**			0.659
Normal	24 (85.7%)	10 (76.9%)	
Increased	4 (14.3%)	3 (23.1%)	
**HGB (g/L)**			0.008
Mean (SD)	126.9 (23.1)	104.2 (22.6)	
Median [MIN, MAX]	133 [75,159]	104 [66,134]	
PLT count (*1000 per uL)			0.007
Mean (SD)	184.2 (45.4)	255.4 (106.7)	
Median [MIN, MAX]	182 [101,264]	243 [65,470]	
NEUT count (*1000 per uL)			0.048
Mean (SD)	3 (1.4)	4.7 (3.1)	
Median [MIN, MAX]	2.6 [1.4,8.3]	3.9 [1,12.7]	
LYMPH count (*1000 per uL)			< 0.001
Mean (SD)	1.7 (0.6)	0.9 (0.3)	
Median [MIN, MAX]	1.7 [0.8,3]	1 [0.3,1.5]	
MONO count (*1000 per uL)			0.274
Mean (SD)	0.4 (0.2)	0.6 (0.4)	
Median [MIN, MAX]	0.4 [0.2,0.9]	0.4 [0.2,1.8]	
**ALB (g/L)**			0.041
Mean (SD)	39.7 (5)	36.5 (4.5)	
Median [MIN, MAX]	40 [25,47.9]	34.9 [29.5,43.9]	
**CD3 count (per ul)**			< 0.001
Mean (SD)	1196.1 (390.2)	646.9 (277.5)	
Median [MIN, MAX]	1167 [428,1844]	606 [159,1039]	
**CD8 count (per ul)**			0.001
Mean (SD)	784.2 (353.9)	402.8 (211.6)	
Median [MIN, MAX]	689 [157,1493]	377 [117,769]	
**CD4 count (per ul)**			0.018
Mean (SD)	373 (221.2)	216.5 (122.9)	
Median [MIN, MAX]	353.5 [21,944]	201 [31,476]	
**CD4/CD8**			0.501
Mean (SD)	0.6 (0.7)	0.7 (0.4)	
Median [MIN, MAX]	0.5 [0.1,3.2]	0.5 [0.1,1.6]	

*AFP* alpha fetoprotein, *CEA* carcinoembryonic antigen, *HGB* hemoglobin, *PLT* platelet, *NEUT* neutrophil, *LYMPH* lymphocyte, *MONO* monocyte, *ALB* albumin

## Discussion

During the era of combination antiretroviral therapy (cART), the incidence of infection-related cancers increased in people living with HIV (PLWH) compared to the general population [4, 5, 19]. The longer life expectancy of PLWH exposed them to chronic infections such as herpes virus 8, human papillomavirus, hepatitis viruses, and *H. pylori*, leading to chronic inflammation and cancer development. However, common epithelial cancers without chronic pathogenic infections did not occur at higher rates, including colorectal, breast, and prostate cancers [4, 5, 20]. *H.pylori* infection was a major risk factor for gastric cancer in the general population, and due to dietary habits, its incidence may be increasing in PLWH in China. Reports suggested that PLWH with gastrointestinal cancer were less likely to receive cancer screening or regular treatment, leading to higher mortality than HIV-negative patients [7, 19]. Furthermore, few studies had examined the association of HIV status with gastric cancer treatment [21]. Our study found that HIV-infected gastric cancer patients had a three-year overall survival rate of only 46%, much lower than the average [3] and GC patients in the general population (Figure S1). Therefore, easily achievable prognostic predictors for patients with HIV-infected GC are needed, so that subgroups of patients with poorer prognoses can be identified earlier, and interventions and follow-up can be initiated. The preoperative systemic inflammation biomarker has proven reliable in various cancers, making it worthwhile to investigate in HIV-positive GC patients.

This study investigated the prognostic value of six systemic inflammation biomarkers (NLR, LMR, PLR, PNI, SII, and SIS) in HIV-infected gastric cancer (GC) patients and compared their prognostic value with non-HIV-infected GC patients at the same center (Table S2 and 3). Previous studies have shown that systemic inflammation biomarkers are useful prognostic factors of gastrointestinal carcinomas [10, 15, 22, 23]. Our results revealed that among surgically treated HIV-infected GC patients, those with high platelet to lymphocyte ratio (PLR) had significantly shorter overall survival (OS) and progression-free survival (PFS) than those with low PLR. Furthermore, multivariate analysis demonstrated that PLR was an independent factor influencing survival outcomes. The findings emphasize the importance of considering systemic inflammation biomarkers, particularly PLR, as prognostic predictors in HIV-infected GC patients.

Lymphocytes play a crucial role in the peritumoral inflammatory response, which reflects the cancer immunosurveillance in the host [24]. Low serum lymphocyte counts have been linked to poor outcomes in patients with gastric cancer (GC) [25]. Platelets, the typical blood cell component responsible for the inflammatory response, are often increased in patients with solid tumors associated with chronic inflammation [26]. In the tumor microenvironment, platelets can interact directly with cancer cells and promote tumorigenesis by releasing pro-angiogenic proteins such as vascular epidermal growth factor and transforming growth factor β [27]. They can also protect circulating tumor cells from host immune surveillance by creating a defense barrier around them [17]. Thus, a higher platelet to lymphocyte ratio (PLR) indicates a greater susceptibility of tumors to immune escape and progression. The PLR has been found to be negatively correlated with patients' survival outcomes in gastric [28], lung [29], urological [30], colorectal [31] and breast cancer [32]. Currently, PLR has significant references for the surgical selection and prognosis of HIV-infected GC patients.

To investigate the correlation between PLR and clinicopathological features, we divided all HIV-infected GC patients into high and low PLR groups according to the cut-off values. The results showed that higher PLR was significantly associated with lower BMI, hemoglobin, and albumin levels (Tables 3 and 4), indicating preoperative malnutrition. This may be one of the reasons explaining the poorer prognosis in the high PLR group. In addition, the high PLR subgroup had a high percentage of postoperative stage III-IV (76.9% vs 50.0%), suggesting a negative correlation between preoperative result of PLR and postoperative disease severity in HIV-infected GC patients (Table 3). Circulating lymphocytes, which are recruited from the bone marrow and differentiate into various types, play a crucial role in accomplishing an anti-tumor response in the tumor microenvironment [33]. In our study, lower PLR was associated with higher peripheral CD4 + and CD8 + T cell counts (Table 4). Moreover, PLR had better predictive value in subgroups with high CD3 + , CD4 + , and CD8 + T cell counts (Figs. 2 and 3). We hypothesized that serum PLR levels may reflect the recovered status of tumor-specific lymphocytes in HIV-infected GC patients, but this needs further research. Furthermore, the high PLR subgroup showed increased neutrophil counts. Neutrophil counts in the circulation were observed to increase during tumor progression [34], but the relationship between neutrophils and PLR in HIV-related GC patients remains unclear.

While this study provides the insights into the prognostic value of PLR in HIV-infected GC patients, there are some limitations that should be considered. Firstly, the relatively small sample size and lack of validation using an independent cohort may have potential for selection bias. It is challenging to collect sufficient number of HIV-related GC cohorts due to the rarity, inadequate knowledge and discriminatory attitudes of this disease. Therefore, collaboration with other HIV treatment centers is crucial to validate the broader applicability of PLR in the future. Secondly, the absence of preoperative C-reactive protein (CRP) data prevented the analysis of CRP-related markers, which may have provided additional prognostic information. Moreover, the lack of data on *H.pylori* infection history limited the exploration of the relationship between inflammation biomarkers and GC exposure factors. Therefore, future studies with larger sample sizes, multi-center, and a more comprehensive analysis of exposure factors are necessary to further elucidate the differences in the prognostic value of inflammation biomarkers in HIV-infected GC patients.

## Conclusion

With the availability of combination antiretroviral therapy (cART), the incidence of gastric cancer (GC) is increasing in individuals infected with human immunodeficiency virus (HIV). Our study demonstrates that preoperative platelet-to-lymphocyte ratio (PLR) can serve as an independent biomarker of overall survival (OS) and progression-free survival (PFS) in HIV-infected GC patients, reflecting their immune status. This simple prognostic biomarker could potentially be used to stratify patients' preoperative risks, which enables personalized treatment for patients with HIV and gastric cancer.

## Supplementary Information

**Additional file 1: Table S1.** Baseline clinicopathologic features and preoperative laboratory tests of GC patients living with HIV. **Table S2.** Univariate and multivariate analysis of clinicopathologic variables in relation to OS in GC patients without HIV infection. **Table S3.** Univariate and multivariate analysis of clinicopathologic variables in relation to PFS in GC patients without HIV infection. **Figure S1.** The Kaplan-Meier survival analysis of Overall Survival and Progression Free Survival between HIV-related and non-HIV related GC patients.

## Data Availability

The datasets used during the current study available from the corresponding author on reasonable request.

## References

[1] Siegel RL, Miller KD, Fuchs HE, Jemal A (2021). Cancer statistics, 2021. CA Cancer J Clin.

[2] Yoshikawa T, Sasako M (2012). Gastrointestinal cancer: Adjuvant chemotherapy after D2 gastrectomy for gastric cancer. Nat Rev Clin Oncol.

[3] Yoshikawa T, Morita S, Tanabe K, Nishikawa K, Ito Y, Matsui T, Fujitani K, Kimura Y, Fujita J, Aoyama T (2016). Survival results of a randomised two-by-two factorial phase II trial comparing neoadjuvant chemotherapy with two and four courses of S-1 plus cisplatin (SC) and paclitaxel plus cisplatin (PC) followed by D2 gastrectomy for resectable advanced gastric cancer. Eur J Cancer.

[4] Grulich AE, van Leeuwen MT, Falster MO, Vajdic CM (2007). Incidence of cancers in people with HIV/AIDS compared with immunosuppressed transplant recipients: a meta-analysis. Lancet.

[5] Yuan T, Hu Y, Zhou X, Yang L, Wang H, Li L, Wang J, Qian H, Clifford GM, Zou H (2022). Incidence and mortality of non-AIDS-defining cancers among people living with HIV: A systematic review and meta-analysis. eClinicalMedicine.

[6] Chen CH, Chung CY, Wang LH, Lin C, Lin HL, Lin HC (2015). Risk of cancer among HIV-infected patients from a population-based nested case-control study: implications for cancer prevention. BMC Cancer.

[7] Coghill AE, Han X, Suneja G, Lin CC, Jemal A, Shiels MS (2019). Advanced stage at diagnosis and elevated mortality among US patients with cancer infected with HIV in the National Cancer Data Base. Cancer-Am Cancer Soc.

[8] Okugawa Y, Toiyama Y, Yamamoto A, Shigemori T, Ichikawa T, Yin C, Suzuki A, Fujikawa H, Yasuda H, Hiro J (2020). Lymphocyte-to-C-reactive protein ratio and score are clinically feasible nutrition-inflammation markers of outcome in patients with gastric cancer. Clin Nutr.

[9] Lin J, Lin J, Xie J, Wang J, Lu J, Chen Q, Cao L, Lin M, Tu R, Zheng C (2019). Prognostic importance of the preoperative modified systemic inflammation score for patients with gastric cancer. Gastric Cancer.

[10] Xin-Ji Z, Yong-Gang L, Xiao-Jun S, Xiao-Wu C, Dong Z, Da-Jian Z (2015). The prognostic role of neutrophils to lymphocytes ratio and platelet count in gastric cancer: a meta-analysis. Int J Surg (London, England).

[11] Kim EY, Lee JW, Yoo HM, Park CH, Song KY (2015). The platelet-to-lymphocyte ratio versus neutrophil-to-lymphocyte ratio: which is better as a prognostic factor in gastric cancer?. Ann Surg Oncol.

[12] Gonzalez-Cao M, Martinez-Picado J, Karachaliou N, Rosell R, Meyerhans A (2019). Cancer immunotherapy of patients with HIV infection. Clin Transl Oncol.

[13] Cao Y, Wu Q, Lian S, Deng L. Lymphocytes Infiltration and expression of PD-1 and PD-L1 in colorectal cancer between HIV-Infected and non-HIV-infected patients: a propensity score matched cohort study. Front Oncol. 2022;12, 10.3389/fonc.2022.827596. 10.3389/fonc.2022.827596 PMC8924460 35311077

[14] Jung MR, Park YK, Jeong O, Seon JW, Ryu SY, Kim DY, Kim YJ (2011). Elevated preoperative neutrophil to lymphocyte ratio predicts poor survival following resection in late stage gastric cancer. J Surg Oncol.

[15] Deng Q, He B, Liu X, Yue J, Ying H, Pan Y, Sun H, Chen J, Wang F, Gao T. et al. Prognostic value of pre-operative inflammatory response biomarkers in gastric cancer patients and the construction of a predictive model. J Transl Med. 2015;13. 10.1186/s12967-015-0409-0. 10.1186/s12967-015-0409-0 PMC4343078 25885254

[16] Toyokawa T, Muguruma K, Yoshii M, Tamura T, Sakurai K, Kubo N, Tanaka H, Lee S, Yashiro M, Ohira M. Clinical significance of prognostic inflammation-based and/or nutritional markers in patients with stage III gastric cancer. BMC Cancer. 2020;20. 10.1186/s12885-020-07010-0. 10.1186/s12885-020-07010-0 PMC7271388 32493247

[17] Hirahara N, Tajima Y, Matsubara T, Fujii Y, Kaji S, Kawabata Y, Hyakudomi R, Yamamoto T, Uchida Y, Taniura T (2021). Systemic immune-inflammation index predicts overall survival in patients with gastric cancer: a propensity score-matched analysis. J Gastrointest Surg.

[18] Nenclares P, Gunn L, Soliman H, Bover M, Trinh A, Leslie I, Wong KH, Melcher A, Newbold K, Nutting CM, et al. On-treatment immune prognostic score for patients with relapsed and/or metastatic head and neck squamous cell carcinoma treated with immunotherapy. J Immunother Cancer. 2021;9. 10.1136/jitc-2021-002718. 10.1136/jitc-2021-002718 PMC8190047 34103355

[19] Robbins HA, Pfeiffer RM, Shiels MS, Li J, Hall HI, Engels EA. Excess cancers among HIV-infected people in the United States. J Natl Cancer Inst. 2015;107. 10.1093/jnci/dju503. 10.1093/jnci/dju503 PMC4334816 25663691

[20] Coghill AE, Engels EA, Schymura MJ, Mahale P, Shiels MS (2018). Risk of Breast, Prostate, and Colorectal Cancer Diagnoses Among HIV-Infected Individuals in the United States. J Natl Cancer Inst.

[21] Pumpalova YS, Segall L, Felli R, Bhatkhande G, Jacobson JS, Neugut AI (2021). The Impact of HIV on Non-AIDS defining gastrointestinal malignancies: a review. Semin Oncol.

[22] Yamamoto T, Kawada K, Obama K (2021). Inflammation-related biomarkers for the prediction of prognosis in colorectal cancer patients. Int J Mol Sci.

[23] Kudou K, Nakashima Y, Haruta Y, Nambara S, Tsuda Y, Kusumoto E, Ando K, Kimura Y, Hashimoto K, Yoshinaga K (2021). Comparison of inflammation-based prognostic scores associated with the prognostic impact of adenocarcinoma of esophagogastric junction and upper gastric cancer. Ann Surg Oncol.

[24] Dunn GP, Old LJ, Schreiber RD. The immunobiology of cancer immunosurveillance and immunoediting. USA: Elsevier Inc. 2004;21:137–48. 10.1016/j.immuni.2004.07.017. 10.1016/j.immuni.2004.07.017 15308095

[25] Eo WK, Jeong DW, Chang HJ, Won KY, Choi SI, Kim SH, Chun SW, Oh YL, Lee TH, Kim YO (2015). Absolute monocyte and lymphocyte count prognostic score for patients with gastric cancer. World J Gastroenterol.

[26] Stone RL, Nick AM, McNeish IA, Balkwill F, Han HD, Bottsford-Miller J, Rupairmoole R, Armaiz-Pena GN, Pecot CV, Coward J (2012). Paraneoplastic thrombocytosis in ovarian cancer. N Engl J Med.

[27] Liu L, Zou J, Guan Y, Zhang Y, Zhang W, Zhou X, Xiong C, Tolbert E, Zhao TC, Bayliss G (2019). Blocking the histone lysine 79 methyltransferase DOT1L alleviates renal fibrosis through inhibition of renal fibroblast activation and epithelial-mesenchymal transition. FASEB J.

[28] Inaoka K, Kanda M, Uda H, Tanaka Y, Tanaka C, Kobayashi D, Takami H, Iwata N, Hayashi M, Niwa Y (2017). Clinical utility of the platelet-lymphocyte ratio as a predictor of postoperative complications after radical gastrectomy for clinical T2–4 gastric cancer. World J Gastroenterol.

[29] Zhang H, Gao L, Zhang B, Zhang L, Wang C (2016). Prognostic value of platelet to lymphocyte ratio in non-small cell lung cancer: a systematic review and meta-analysis. Sci Rep.

[30] Li DY, Hao XY, Ma TM, Dai HX, Song YS (2017). The prognostic value of platelet-to-lymphocyte ratio in urological cancers: a meta-analysis. Sci Rep.

[31] Huang XZ, Chen WJ, Zhang X, Wu CC, Zhang CY, Sun SS, Wu J (2017). An elevated platelet-to-lymphocyte ratio predicts poor prognosis and clinicopathological characteristics in patients with colorectal cancer: a meta-analysis. Dis Markers.

[32] Zhang M, Huang XZ, Song YX, Gao P, Sun JX, Wang ZN (2017). High platelet-to-lymphocyte ratio predicts poor prognosis and clinicopathological characteristics in patients with breast cancer: a meta-analysis. Biomed Res Int.

[33] De Palma M, Biziato D, Petrova TV (2017). Microenvironmental regulation of tumour angiogenesis. Nat Rev Cancer.

[34] Ustyanovska AN, Visser N, Bremer E, Wiersma VR. The neutrophil: the underdog that packs a punch in the fight against cancer. Int J Mol Sci. 2020;21. 10.3390/ijms21217820. 10.3390/ijms21217820 PMC7659937 33105656

