# Supplementary material for: Preoperative Platelet-Lymphocyte Ratio (PLR) as a prognostic inflammation biomarker in Asian HIV-infected patients with gastric cancer: a single-center study
Source: BMC Gastroenterol. 2023 May 26;23:187. doi: 10.1186/s12876-023-02828-x (PMC10214675; doi:10.1186/s12876-023-02828-x)
Supplement: Supplementary file 1 — Additional file 1: Table S1. Baseline clinicopathologic features and preoperative laboratory tests of GC patients living with HIV. Table S2. Univariate and multivariate analysis of clinicopathologic variables in relation to OS in GC patients without HIV infection. Table S3. Univariate and multivariate analysis of clinicopathologic variables in relation to PFS in GC patients without HIV infection. Figure S1. The Kaplan-Meier survival analysis of Overall Survival and Progression Free Survival between HIV-related and non-HIV related GC patients. [file 12876_2023_2828_MOESM1_ESM.zip › Figure S1.docx]

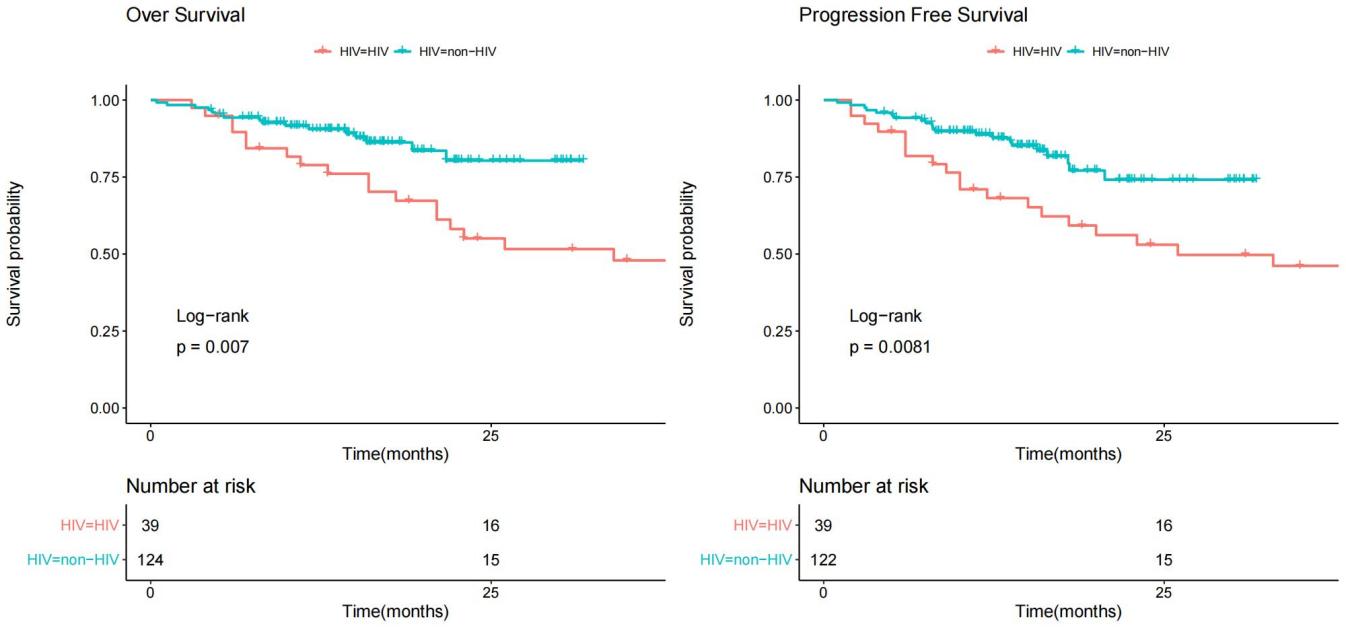


Figure S1: The Kaplan-Meier survival analysis of Overall Survival and Progression Free Survival between HIV-related and non-HIV related GC patients.
